# Genetic Connection between Hyperglycemia and Carotid Atherosclerosis in Hyperlipidemic Mice

**DOI:** 10.3390/genes13030510

**Published:** 2022-03-14

**Authors:** Lisa J. Shi, Bilhan Chagari, Alexander An, Mei-Hua Chen, Yongde Bao, Weibin Shi

**Affiliations:** 1 Department of Radiology and Medical Imaging, University of Virginia, Charlottesville, VA 22908, USA; lisajshi@utexas.edu (L.J.S.); bc8he@virginia.edu (B.C.); aca9kr@virginia.edu (A.A.); mc2xc@hscmail.mcc.virginia.edu (M.-H.C.); 2 Department of Microbiology, University of Virginia, Charlottesville, VA 22908, USA; yb8d@virginia.edu; 3 Department of Biochemistry and Molecular Genetics, University of Virginia, Charlottesville, VA 22908, USA

**Keywords:** atherosclerosis, carotid artery, quantitative trait locus, type 2 diabetes, hyperlipidemia

## Abstract

Type 2 diabetes (T2D) is a major risk for atherosclerosis and its complications. *Apoe*-null (*Apoe*^−/−^) mouse strains exhibit a wide range of variations in susceptibility to T2D and carotid atherosclerosis, with the latter being a major cause of ischemic stroke. To identify genetic connections between T2D and carotid atherosclerosis, 145 male F2 mice were generated from LP/J and BALB/cJ *Apoe*^−/−^ mice and fed 12 weeks of a Western diet. Atherosclerotic lesions in the carotid arteries, fasting, and non-fasting plasma glucose levels were measured, and genotyping was performed using miniMUGA arrays. Two significant QTL (quantitative trait loci) on chromosomes (Chr) 6 and 15 were identified for carotid lesions. The Chr15 QTL coincided precisely with QTL *Bglu20* for fasting and non-fasting glucose levels. Carotid lesion sizes showed a trend toward correlation with fasting and non-fasting glucose levels in F2 mice. The Chr15 QTL for carotid lesions was suppressed after excluding the influence from fasting or non-fasting glucose. Likely candidate genes for the causal association were *Tnfrsf11b*, *Deptor*, and *Gsdmc2*. These results demonstrate a causative role for hyperglycemia in the development of carotid atherosclerosis in hyperlipidemic mice.

## 1. Introduction

Atherosclerosis is a chronic inflammatory disease of large and medium-sized arteries, such as the coronary artery and carotid artery, featured by the buildup of lipid-containing plaque in the arterial wall. Plaque enlarges and ruptures the narrow arterial lumen and obstructs blood flow to the brain, heart, and other organs, leading to stroke, heart attack, and other adverse complications [1]. Stroke is the fourth most common cause of death and the leading cause of disability in adults of the United States [2]. Ischemic stroke, resulting from obstruction of blood flow to the brain, accounts for 87% of all strokes. A large fraction of ischemic stroke cases is caused by atherosclerosis in the carotid arteries [3]. Atherosclerosis is a highly heritable disorder affected by multiple genes as well as environmental factors. Heritability estimates for carotid intima-media thickness (cIMT) and carotid plaque are high, with some estimates exceeding 50% [4,5]. The latest meta-analysis of genome-wide association studies (GWAS) with Europeans from 17 studies identified 14 loci for cIMT and carotid plaque [6]. However, these loci only account for a small fraction of the variance in the traits of the examined subjects. Moreover, the effect sizes of the loci detected by GWAS are generally small [7], so identification of the underlying causal variants is extremely challenging. Therefore, parallel approaches need to be undertaken to facilitate identification of genes for carotid atherosclerosis by using animal models.

Phenotypically diverse mouse strains provide a powerful experimental system for mapping and functional analysis of genes contributing to human health and disease [8]. Almost all of the genes in mice share functions with the genes in humans, and the two species are highly comparable in development, physiology, and genome organization [9,10]. However, wild-type mice do not develop atherosclerosis in the carotid artery [11,12], and thus preclude their use for genetic study of carotid atherosclerosis. *Apoe*-null (*Apoe*^-/--/-^) mice develop spontaneous hyperlipidemia and atherosclerosis on a rodent chow diet, which are accelerated by feeding a high fat diet. As seen in humans, atherosclerotic lesions in *Apoe*^-/--/-^ mice develop at branch points of large and medial arteries and progress from fatty streak to advanced plaque with fibrous caps and necrotic lipid core [13]. We constructed multiple *Apoe*^−/−^ mouse strains with various genetic backgrounds [14] and used them to perform quantitative trait locus (QTL) analysis of carotid atherosclerosis. Twelve significant QTLs, named *Cath1* through *Cath12*, have been mapped for carotid atherosclerosis from three independent crosses derived from *Apoe*^−/−^ mouse strains [15,16,17,18]. Additional crosses need to be generated to find more QTLs and genes for carotid atherosclerosis.

Type 2 diabetes (T2D) is a major risk factor for atherosclerosis and its complications, including ischemic stroke. A meta-analysis of ~700,000 patients from 102 prospective studies has shown that patients with diabetes had two- to three-times higher risk of developing ischemic stroke [19]. A shared genetic basis, such as pleiotropy and linkage disequilibrium, has been proposed to explain the co-occurrence of T2D and atherosclerosis [20]. Genetic evidence supporting the theory is that SNPs (single nucleotide polymorphisms) robustly associated with T2D in GWAS have shown an increased association with coronary heart disease (CHD) [21,22]. However, no such enrichment in association with T2D has been found for SNPs significantly associated with CHD [22]. Even though a SNP is found to be associated with both T2D and CHD, it is challenging to deduce their causal connections from human GWAS due to the small effect size of an individual variant, complex genetic structure, and environmental influences.

*Apoe*^−/−^ mice on certain genetic backgrounds develop T2D when fed a Western diet [14,23], thus providing a valuable model for finding common genetic loci shared between T2D and carotid atherosclerosis. When shared genetic loci for two traits are found, the causal effect of one trait on another can be assessed using causal inference methods [24]. In the present study, we sought to determine whether T2D plays a causal role in the development of carotid atherosclerosis.

## 2. Materials and Methods

### 2.1. Mice

BALB/cJ (BALB) and LP/J (LP) *Apoe*^−/−^ mice were generated in our laboratory using the classic breeding scheme, as reported in [14]. LP-*Apoe*^−/−^ males were crossed with BALB-*Apoe*^−/−^ females to generate F1s, which were intercrossed to generate a male F2 cohort. Mice were weaned at three weeks of age onto a chow diet, started at six weeks of age with a Western diet containing 21% fat, 34.1% sucrose, 0.15% cholesterol, and 19.5% casein (TD 88137, Envigo, Indianapolis, IN, USA), and kept on the diet for twelve weeks. Non-fasting blood was collected after mice were fed 11 weeks of Western diet, and fasting blood was collected after 12 weeks on the Western diet. A one-week interval was needed between two bleedings, according to an approved animal protocol. Mice were fasted overnight before fasting blood was collected and body weight measured at the time of being euthanized. All blood samples were drawn from the retro-orbital veins, with the animals being anesthetized by isoflurane inhalation using a heparin-coated microcapillary tube and collected into a 1.5-mL eppendorf tube containing 8 μL of 0.5 M ethylenediaminetetraacetic acid (EDTA). After a five-min centrifugation at 13,000× *g* at 4 °C, the plasma fraction was collected and stored at −80 °C before assay. All procedures were carried out according to current National Institutes of Health guidelines and approved by the institutional animal care and use committee (protocol #: 3109).

### 2.2. Measurement of Atherosclerotic Lesions

Atherosclerotic lesions in the left common carotid bifurcation were quantified as reported [16,17,18]. Briefly, the vasculature of mice was first flushed with saline and then perfusion-fixed with 10% formalin through the heart. The distal portion of the common carotid artery and adjacent branches was dissected en bloc and embedded in Tissue-Tek optimum cutting compound. Ten-μm-thick cryosections were collected every three sections, stained with oil red O and hematoxylin, and counterstained with light green. Lesion sizes were measured using Zen 3.4 imaging software. Results on five sections with the largest readings were averaged for each mouse, and this average was used for statistical analysis.

### 2.3. Glucose Assay

Plasma glucose levels were measured with a Sigma assay kit (Cat. # GAHK20, Saint Louis, MO, USA), an assay based on the hexokinase oxidase reaction, as we reported [25]. In brief, 6 μL of diluted plasma samples (3× in water), together with standards and controls, were loaded in a 96-well plate and mixed with 150 µL of assay reagent per well. After a 30-min incubation at 30 °C, the absorbance at 340 nm was measured with a Molecular Devices plate reader.

### 2.4. Genotyping

DNA was prepared from the tails of mice using QIAGEN kits (San Diego, CA, USA). Genotyping was performed at Neogen (Lansing, MI, USA) using the miniMUGA array, which contains 11,000 SNP probes built on an Illumina Infinium platform. Parental and F1 DNA served as controls on each array. Uninformative markers were excluded from QTL analysis. Informative SNP markers were filtered based on the expected genotyping results of the control samples. Possible genotyping errors were further checked using the “calc errorlod” function of R/qtl software, Version 1.50. A total of 2595 SNPs passed quality control and were used for QTL mapping.

### 2.5. Statistical Analysis

QTL mapping was performed using R/qtl and Map Manager QTXb17, as reported in [26,27,28]. To define genome-wide LOD (logarithm of odds) score thresholds for significant and suggestive linkage with each trait, 1000 permutations were run at a 1-Mb interval across the genome. Loci with a genome-wide *p* value of <0.05 were deemed to be significant, and those with a genome-wide *p* value of <0.63 were suggestive [29]. The allele effect of each QTL was determined by calculating the phenotype means and SD for each of the three possible genotypes.

SNP markers on the miniMUGA array are spaced at ~0.25 Mb across the genome. Thus, adjacent markers may share identical genotyping results across all the F2 mice because recombination segments in the second generation of offspring are often longer than a few Mb [30]. For interval mapping analysis with QTX, redundant markers needed to be excluded so that each marker had a unique genotype for the F2 cohort.

### 2.6. Establishment of Causal Relationship between Traits Using Overlapping QTL

When overlapping QTL for two traits were detected, additional analysis was performed to infer causal relationships between the traits, as previously described [26,31]. Briefly, residuals were generated from regression analysis of two affected traits and then subject to genome-wide QTL mapping with the same algorithm previously used for the identification of the overlapped QTL. The QTL yielded from the residual variation in one trait would be independent of variation in another.

### 2.7. Prioritization of Candidate Genes

Bioinformatics resources were used to prioritize candidate genes for significant QTL that had been mapped in two or more crosses derived from different parental strains whose genome sequence and variant data were available. Variants were queried for through the Sanger Mouse Genomes Project (https://www.sanger.ac.uk/sanger/Mouse_SnpViewer/rel-1505, 14 February 2022). Probable candidate genes were those containing one or more missense SNPs or SNP(s) in upstream regulatory regions that co-segregated between high and low alleles at QTL, as described in [18,32,33]. The SIFT (Sorting Intolerant from Tolerant) score was obtained through the Ensembl Genome Browser (https://useast.ensembl.org/index.html, 14 February 2022) and used for predicting the effect of a missense variant on protein function [34].

## 3. Results

### 3.1. Trait Value Frequency Distributions

**Figure 1:**
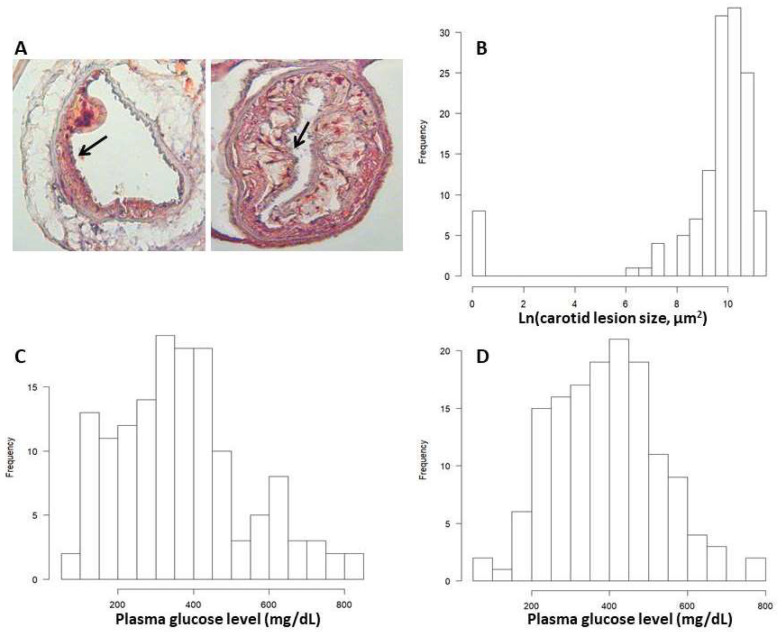
Representative images of carotid atherosclerosis (**A**) and trait value distributions of log-transformed carotid lesion sizes (**B**), fasting (**C**) and non-fasting plasma glucose levels (**D**) of F2 mice. Sections were stained with oil red O. Arrows point at atherosclerotic lesions. The bar graphs were created with a plot function of R/qtl.

Atherosclerotic lesions in the left carotid arteries of F2 mice fed 12 weeks of Western diet were measured after being stained with oil red O. Of the 145 F2 mice, 129 (94.5%) formed atherosclerotic lesions in the vessels, 8 mice (5.5%) developed no lesion, and 8 had missing data (Figure 1). Values of log-transformed carotid lesion sizes exhibited a bimodal distribution: the single rectangle bar on the left edge represents F2 mice that had no lesion, and the bell-shaped histogram on the right represents mice with various sizes of carotid lesions. Values of fasting and non-fasting plasma glucose levels were approximately normally distributed.

### 3.2. Validating the Effectiveness of the F2 Cohort: Mapping the Albino Locus

**Figure 2:**
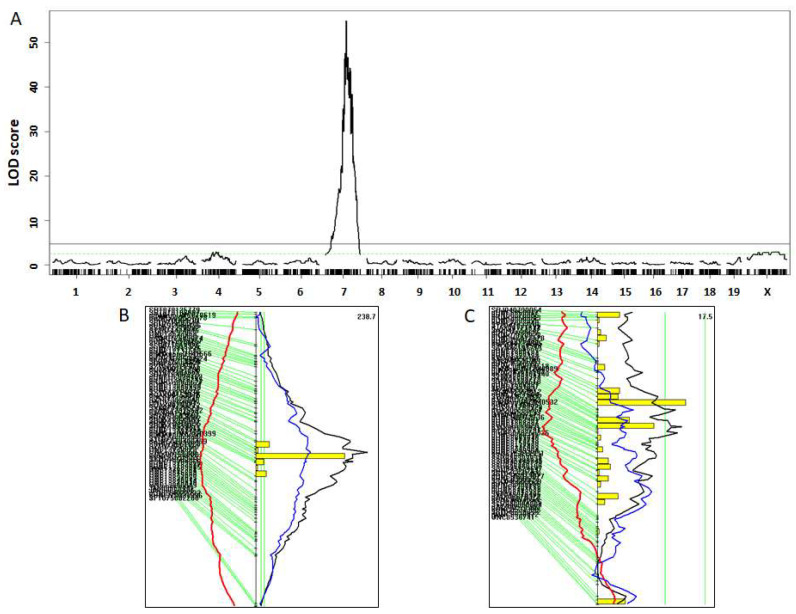
Testing the reliability of the F2 cohort derived from BALB-*Apoe*^−/−^ and LP-*Apoe*^−/−^ mice via mapping the albino coat color locus. (**A**) Genome-wide scan to detect a huge albino locus to chromosome 5 at 88.1 Mb. The X axis shows the chromosomal position and the Y axis shows the LOD score. Two horizontal lines represent the genome-wide thresholds for significant linkage at *p* = 0.05 (black) and suggestive linkage at *p* = 0.63 (green). (**B**) Interval mapping plot for chromosome 7 harboring the huge albino locus. The curved black line denotes LOD score calculated at a 1-Mb interval along the chromosome. The blue and red lines denote dominant and additive regression coefficients, respectively. Yellow histograms denote the confidence interval estimated by the bootstrap test. Two vertical green lines denote genome-wide significance thresholds at *p* = 0.63 and *p* = 0.05, respectively. Genetic markers used are shown on the left of the figure. (**C**) Interval mapping plot for chromosome 4 harboring a suggestive locus for coat color.

The two parental strains are distinct in fur color, with BALB mice being albino and LP mice agouti. A missense mutation of *Tyr* (87.1 Mb), encoding the tyrosinase on chromosome (Chr) 7, is deemed responsible for the albino fur color of BALB mice [35]. F2 mice displayed a few fur colors, varying from albino to light brown to agouti. We graded the fur colors to three levels: 0 for albino, 1 for light brown, and 2 for agouti. QTL analysis on the F2 mice revealed a huge QTL on Chr7 and a suggestive QTL on Chr4 for fur color (Figure 2). The Chr7 QTL had an extremely high LOD score of 54.9 and a narrow confidence interval between 87.1 and 88.1 Mb (Table 1), where *Tyr* sits. F2 mice homozygous for the BALB allele had the BABL phenotype (white fur), while those homozygous for the LP allele or heterozygous for both BALB and LP alleles had the LP phenotype (agouti color) at the locus. Altogether, 27.7% of the F2 mice were albino, 13.5% were light brown and 58.9% were agouti (Supplemental Data). The observed 27.7% of the F2 mice having white fur is consistent with the expected proportion of ¼, at which the mutant *Tyr* gene from the BALB allele affects fur color in a recessive mode of inheritance.

The Chr4 QTL had a suggestive LOD score of 2.90 and peaked at 77.8 Mb (Figure 2). This QTL replicates *Chop2*, mapped using the collaborative cross developed through a community effort [36]. *Tyrp1*, encoding the tyrosinase-related protein, is the likely causal gene of *Chop2*.

### 3.3. Carotid Atherosclerosis

**Figure 3:**
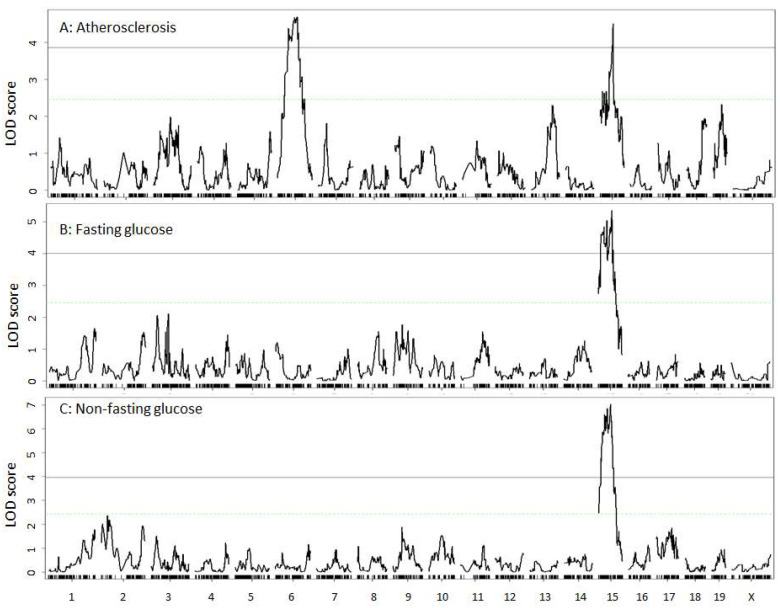
Genome-wide scans to search for loci influencing carotid atherosclerosis (**A**), fasting plasma glucose (**B**), and non-fasting glucose levels (**C**). Chromosomes 1 through X are represented on the X axis. Each short vertical bar on the X axis represents a SNP marker. The Y axis represents the LOD score. The two horizontal lines represent the genome-wide thresholds for significant (black) and suggestive linkage (green).

Two significant QTLs on Chr6 and Chr15 were detected for atherosclerotic lesion sizes (Figure 3A). Details of these QTLs, including locus name, LOD score, 95% confidence interval, peak location, high allele, mode of inheritance, and allelic effect are presented in Table 1. The Chr6 QTL had a significant LOD score of 4.70 and peaked at 86.7 Mb. F2 mice homozygous for the LP allele had larger lesion sizes than those homozygous for the BALB allele at the locus (Table 1). This QTL replicates *Cath4*, previously mapped in B6 × C3H and B6 × BALB *Apoe*^−/−^ intercrosses [15,16].

The Chr15 QTL had a significant LOD score of 4.51 and peaked at 57.3 Mb. This QTL partially overlaps in the confidence interval with *Cath5*, initially mapped in a BALB × SM *Apoe*^−/−^ intercross [17]. The LP allele was responsible for larger lesion size, while the BALB allele decreased lesion size at the locus (Table 1).

### 3.4. Fasting and Non-Fasting Plasma Glucose Levels

Fasting plasma glucose levels of F2 mice were significantly lower than non-fasting glucose levels (368 ± 168 vs. 392 ± 132 mg/dL; *p* = 0.034). A significant QTL on Chr15 was identified for both fasting and non-fasting plasma glucose levels (Figure 3B,C). For fasting glucose, the QTL had a significant LOD score of 5.36 and peaked at 59.7 Mb. For non-fasting glucose, the QTL had a significant LOD score of 7.03 and peaked at 53.3. The LP allele increased and the BALB allele lowered plasma glucose levels (Table 1). This QTL overlaps with *Bglu8*, mapped in a NZB/B1NJ × NZW/LacJ intercross [37], and *Dbm4*, mapped in Akita × A/J F2 mice [38]. We named this QTL *Bglu20* as it was mapped in a cross derived from different parental strains in accordance to the guideline provided by the International Committee on Standardized Genetic Nomenclature for Mice (http://www.informatics.jax.org/mgihome/nomen/gene.shtml, 14 February 2022).

### 3.5. Coincident QTL for Carotid Atherosclerosis and Plasma Glucose

**Figure 4:**
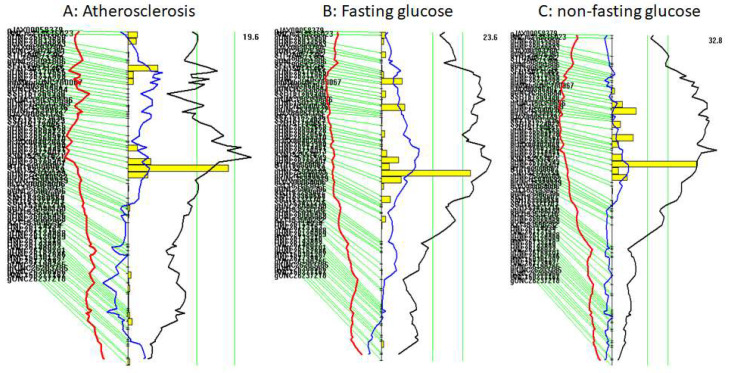
Interval mapping plots for carotid atherosclerosis (**A**), fasting plasma glucose (**B**), and non-fasting glucose (**C**) on chromosome 15. Plots were created using the interval mapping function of Map Manager QTX. The curved black line denotes LOD score calculated at a 1-Mb interval along the chromosome. The red and blue lines denote additive and dominant regression coefficients, respectively. The yellow histograms denote confidence intervals estimated through the bootstrap test. Two vertical green lines denote genome-wide significance thresholds at *p* = 0.63 and *p* = 0.05, respectively. Genetic markers used are shown on the left of the figure.

Interval mapping graphs for Chr15 show that QTL for atherosclerosis (*Cath5*) colocalized with QTL for fasting and non-fasting glucose (*Bglu20*) levels (Figure 4). The LP allele was associated with increased atherosclerotic lesion size and elevated plasma levels of glucose, while the BALB allele had opposite effects on these traits (Table 1).

### 3.6. Associations of Atherosclerotic Lesion Sizes with Plasma Glucose Levels

**Figure 5:**
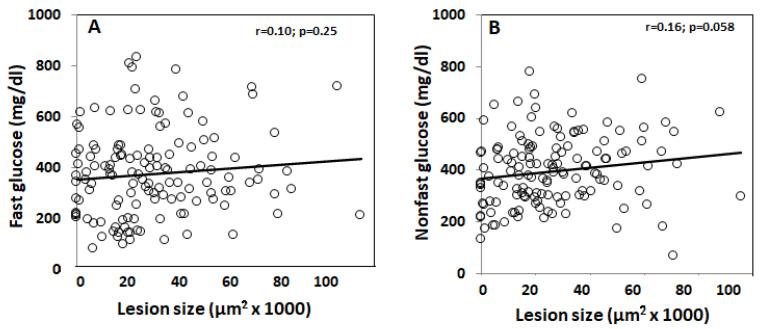
Associations of carotid lesion sizes with fasting (**A**) and non-fasting plasma glucose levels (**B**) among male F2 mice. Each circle represents values of an individual F2 mouse. The correlation coefficient (*r*) and significance (*p*) are shown.

Associations of atherosclerotic lesion sizes with plasma glucose levels were analyzed using F2 mice. Carotid lesion sizes showed a trend toward association with fasting (*r* = 0.10; *p* = 0.25) and non-fasting glucose levels (*r* = 0.16; *p* = 0.058) (Figure 5A,B). F2 mice with higher glucose levels tended to have larger lesion sizes.

### 3.7. Causal Association between Atherosclerosis and Hyperglycemia

**Figure 6:**
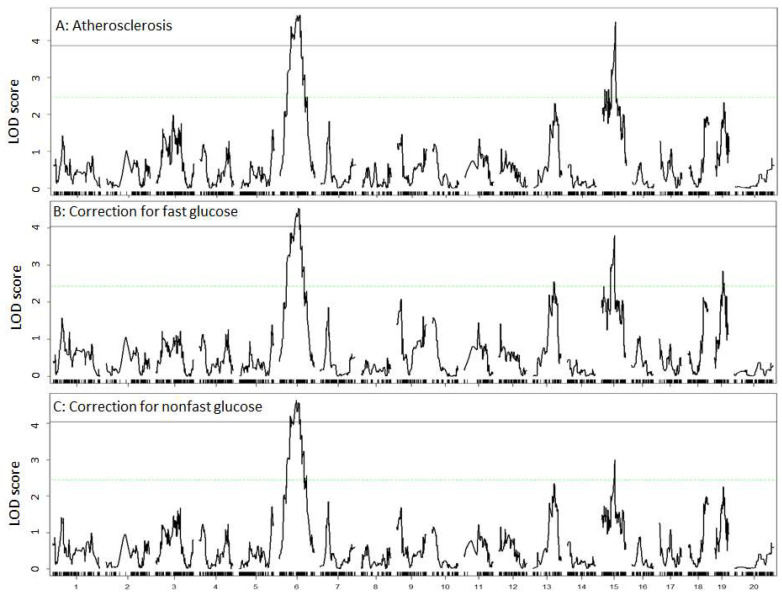
Genome-wide scans to assess the dependence of QTL for carotid atherosclerosis (**A**) on fasting (**B**) and non-fasting plasma glucose levels (**C**) in F2 mice. Residuals from the linear regression analysis of carotid lesion sizes with plasma glucose levels were subject to genome-wide scans. Note the reduced magnitude of the Chr15 QTL for carotid atherosclerosis after correction for fasting and non-fasting glucose.

Since the QTL for atherosclerotic lesions was overlapping with the QTL for plasma glucose levels on Chr15, we examined potential causal associations between the traits. Residuals generated from linear regression analysis of carotid lesion sizes with either fasting or non-fasting glucose in F2 mice were subject to QTL mapping as a new phenotype. When the residuals from regression analysis with fasting or non-fasting glucose levels were analyzed, the Chr15 QTL for atherosclerosis showed a reduced LOD score (3.8 for fasting glucose, 3.0 for non-fasting glucose) (Figure 6B,C), implying a causal association between the two traits.

### 3.8. Prioritization of Candidate Genes

QTL for plasma glucose levels on Chr15 were also mapped in a NZB/B1NJ × NZW/LacJ intercross [37], a KK/Ta × (BALB/c × KK/Ta) backcross [39], and an Akita × A/J intercross [38]. At the QTL, the NZB/B1NJ, KK/Ta, and LP alleles were associated with higher plasma glucose levels, while the NZW/LacJ, BALB, and A/J alleles had opposite effects on the trait. Ten genes within the 45–75 Mb congenic region contained one or more missense SNPs or SNP(s) in upstream regulatory regions that were shared by two or more high allele strains, but are different from those shared by two low allele strains (Table 2). These genes include *Ext1*, *Samd12*, *Tnfrsf11b*, *Colec10*, *Mal2*, *Enpp2*, *Deptor*, *Gsdmc2*, *Gsdmc3*, and *Gsdmc4*. Of them, *Tnfrsf11b*, *Deptor*, and *Gsdmc2* contained one or more missense variants with a low SIFT score, predicted to affect protein function.

## 4. Discussion

In this study, we identified two significant QTLs on mouse chromosomes 6 and 15 for carotid atherosclerosis and a significant QTL on chromosome 15 for plasma glucose levels using a male F2 cohort derived from LP and BALB *Apoe*^−/−^ mice. We observed the colocalization of the QTL for carotid atherosclerosis with the QTL for plasma glucose on chromosome 15. Moreover, the QTL for carotid atherosclerosis on chromosome 15 was suppressed after adjustment for fasting and non-fasting glucose levels.

BALB and LP are among the common mouse strains whose genomes have been sequenced [40]. Thus, available sequence variant data allow for ready identification of candidate genes when QTLs for complex traits or diseases are mapped in crosses derived from the strains. The two strains are distinct in their fur color, and the F2 mice exhibited a few fur colors, including white, light brown, and agouti. A point mutation in the *Tyr* gene, encoding tyrosinase, is responsible for the albino fur color of BALB mice [36]. To validate the effectiveness of the F2 cohort in QTL mapping, we graded their fur colors and conducted QTL analysis of the trait. A huge QTL (LOD: 54.9) maps to Chr7: 87.1–88.1 Mb, with the BALB allele being linked to albino fur color. The *Tyr* gene lies at 87.1 Mb and falls within the 87.1–88.1 Mb confidence interval of the QTL. The observed 27.7% of the F2 mice with a white fur is consistent with the expected proportion of 25% when the mutant *Tyr* gene confers the white fur color in a recessive mode of inheritance.

An intriguing finding of this study is that the QTL for carotid atherosclerosis (*Cath5*) colocalized with the QTL for plasma glucose (*Bglu20*) on chromosome 15. This colocalization provided an opportunity for elucidating causal relationships between the closely related traits. Using a causal inference test by subtracting the biological variation in one trait from the other and using the residual variation for QTL analysis of the other trait, we demonstrated that both fasting and non-fasting glucose levels have a direct influence on atherosclerotic lesion sizes. Indeed, after adjustment for the traits, the Chr15 QTL for atherosclerosis showed reductions in LOD score, i.e., reduced allelic effect on atherosclerotic lesion sizes. As fasting and non-fasting hyperglycemias are the defining features of diabetes, the current finding indicates that diabetes increases the risk for atherosclerosis and its complication ischemic stroke by enhancing plaque growth.

A trend of correlation between atherosclerotic lesions and plasma glucose levels was observed in the F2 cohort under both fasting and non-fasting conditions. With regards to the complex nature of both traits influenced by many common genetic variants, with each having small effects, this result would be considered biologically significant. Interestingly, the strength of a causal association inferred from the overlapped QTL on chromosome 15 is consistent with the correlation coefficient we observed between the affected traits. Indeed, non-fasting plasma glucose levels showed a larger causal association with atherosclerotic lesion sizes, so a trend toward a closer correlation was observed between the traits.

QTLs for plasma glucose on chromosome 15 have been mapped in multiple crosses, including this cross, a NZB/B1NJ × NZW/LacJ intercross [37], a KK/Ta × (BALB/c × KK/Ta) backcross [39], and an Akita × A/J intercross [38]. Using the mapping and available sequence variant data, we prioritized 10 candidate genes, all of which contained one or more missense SNPs or SNP(s) in upstream regions segregating between the high allele and low allele strains. As 97% of the genetic variants between common mouse strains are ancestral [41], QTL genes are almost certainly those containing polymorphisms shared among mouse strains. *Tnfrsf11b*, *Deptor*, and *Gsdmc2* are top candidate genes, with each containing one or more missense SNPs that are predicted to impact protein function. *Deptor* polymorphisms have been shown to be associated with lipid metabolism and risk for macrovascular and microvascular complications in patients with type 2 diabetes [42].

We previously reported that *Apoe*^−/−^ mice on certain genetic backgrounds develop type 2 diabetes when fed a Western diet [14]. Mice with a fasting plasma glucose level exceeding 250 mg/dL are considered diabetic [43]. Thus, a large proportion of the F2 mice developed type 2 diabetes on the Western diet. As seen in humans [44], non-fasting glucose levels were significantly higher than fasting levels in F2 mice. Postprandial glucose levels have been shown to be a better predictor of cardiovascular events and/or all-cause mortality than fasting blood glucose in non-diabetic cohorts or general populations [45,46]. Accordingly, we observed that non-fasting plasma glucose is more closely correlated with carotid lesion sizes than fasting glucose, and the causal inference test showed a closer causal association of non-fasting plasma glucose with carotid atherosclerosis.

A significant QTL for carotid atherosclerosis maps to Chr6: 86.7 Mb, with the LP allele increasing lesion sizes. This QTL replicates Cath4, mapped in B6 × C3H and B6 × BALB *Apoe*^−/−^ intercrosses [15,16]. As it is mapped in multiple crosses derived from different inbred strains, we used available sequence variant data on the parental strains to prioritize candidate genes for Cath4. *Sspo*, *Gimap8*, and *Stk31* were identified as the most likely candidate genes, with each possessing one or more intolerant missense variants that are predicted to affect protein function (Supplemental data).

In summary, we have mapped multiple QTLs for carotid atherosclerosis and plasma glucose levels and demonstrated the causal connections of fasting and non-fasting glucose with atherosclerotic lesion sizes in a segregating F2 cohort. Using combined QTL mapping and all available bioinformatics resources, we have prioritized a few likely candidate genes underlying the genetic connection between type 2 diabetes and carotid atherosclerosis. Nevertheless, functional study is needed to further validate the candidate genes. As complications of atherosclerosis are the leading causes of mortality among patients with type 2 diabetes, these genes, once confirmed, can be valuable targets for developing new treatments for diabetic macrovascular disease. Insulin resistance and associated reductions in cardiac insulin metabolic signaling are major factors for the development of heart failure [47]. Western diet-induced hyperglycemia and hyperlipidemia are major drivers of oxidative stress and systemic inflammation [26,48], which are major factors contributing to the development of cardiac insulin resistance [47]. Thus, it is intriguing to speculate that cardiac insulin resistance may act beyond changes in the plasma glycemic state during the development of type 2 diabetes-accelerated atherosclerosis. This study has the following limitations: first, only male mice were included. QTLs for atherosclerosis mapped from female mice are often distinct from those mapped from males, even from the same cross [49,50]. Second, no transcriptome analysis that could identify eQTLs and additional candidate genes was performed. Finally, the current haplotype analysis targeted candidate genes with missense SNPs and SNPs in upstream regulatory regions. Thus, candidates with variants in introns and downstream regulatory regions as well as 3′ UTR regions that may affect mRNA turnover could be missed.

## Figures and Tables

**Table 1:** Significant and suggestive QT for carotid atherosclerosis, plasma glucose, body weight, and coat color mapped with male F2 mice derived from LP- and BALB-*Apoe*^−/−^ mice.

Locus Name	Chr	LOD ^a^	Peak (Mb)	Closest Marker	95%CI (Mb) ^b^	High Allele	Mode of Inheritance	Allelic Effect ^c^		
								BB	H	LL
Carotid lesion										
*Cath4*	6	**4.70**	86.7	c6.loc81	48.7–92.6	LL	Additive	19023 ± 19176	24111 ± 17454	40596 ± 20223
***Cath5***	15	**4.51**	57.3	gUNC25658310	13.7–64.9	LL	Additive	13805 ± 13652	27306 ± 19074	34455 ± 21065
Glucose (non-fast)										
*Bglu8*, *Fbgl2*, *Dbm4*	15	**7.03**	53.3	gUNC25604126	21.3–58.1	LL	Additive	290 ± 92	392 ± 128	461 ± 127
Glucose (fast)										
*Bglu20*, *Bglu8*, *Fbgl2*, *Dbm4*	15	**5.36**	59.7	c15.loc56	13.7–64.9	LL	Additive	260 ± 116	397 ± 161	420 ± 178
Coat color										
*Choq2*	4	2.90	77.8	gUNC7746435	50.8–128.8	LL	Recessive	1 ± 0.7	1 ± 0.9	2 ± 0.8
***Albc2***	7	**54.9**	88.1	c7.loc85	87.1–88.1	LL	Dominant	0 ± 0	2 ± 0.4	2 ± 0.4
*-*	20	2.86	132.0	UNC31325940	32.8–158.9	LL	-	1.3 ± 0.9	-	1.4 ± 0.9

^a^ LOD scores were obtained from genome-wide QTL analysis using R/qtl. Significant QTL and LOD scores were highlighted in bold. ^b^ 95% Confidence interval in Mb for significant or suggestive QTL. ^c^ BB: BALB allele; LL: LP allele; H: Heterozygous for both BALB and LP alleles. Unit for carotid lesion: µm^2^; plasma glucose: mg/dL; for body weight: g; for coat color: grade. Values for allelic effect were expressed as means ± SD.

**Table 2:** Positional candidate genes for *Bglu20* on chromosome 15 identified by haplotype analysis.

Chr	Position	Gene	dbSNP	Ref	LP_J	KK_HiJ	NZB_B1NJ	BALB_cJ	NZW_LacJ	A_J	Csq	AA	AA Coord	SIFT Score
15	53346329	Ext1	rs251984497	A	C	C	-	-	-	C	Upstream variant			
15	53350791	Ext1	rs50212623	G	A	A	-	-	-	A	Upstream variant			
15	53902704	Samd12	rs31898388	A	G	G	-	-	-	G	Upstream variant			
15	53903266	Samd12	rs31558297	T	C	C	-	-	-	C	Upstream variant			
15	53903360	Samd12	rs31587200	T	C	C	-	-	-	C	Upstream variant			
15	53904866	Samd12	rs31667666	T	C	C	-	-	-	C	Upstream variant			
15	53905089	Samd12	rs32330337	A	G	G	-	-	-	G	Upstream variant			
15	53905527	Samd12	rs47273772	T	C	C	-	-	-	C	Upstream variant			
15	53906558	Samd12	rs31763540	T	C	C	-	-	-	C	Upstream variant			
15	53906921	Samd12	rs31682534	A	G	G	-	-	-	G	Upstream variant			
15	54252313	Tnfrsf11b	rs31799791	A	C	-	-	-	-	C	Missense variant	L/R	296	0.61
15	54252338	Tnfrsf11b	rs32100171	A	C	-	-	-	-	C	Missense variant	S/A	288	1
15	54256095	Tnfrsf11b	rs51638693	A	C	-	-	-	-	C	Missense variant	I/R	161	1
15	54256164	Tnfrsf11b	rs33484516	C	T	-	-	-	-	T	Missense variant	R/Q	138	0.23
15	54278530	Tnfrsf11b	rs47057076	G	T	-	-	-	-	T	Upstream variant			
15	54278620	Tnfrsf11b	rs49814729	A	G	-	-	-	-	G	Upstream variant			
15	54278628	Tnfrsf11b	rs33490015	A	G	-	-	-	-	G	Upstream variant			
15	54278736	Tnfrsf11b	rs33489243	G	C	-	-	-	-	C	Upstream variant			
15	54278937	Tnfrsf11b	rs51583114	T	C	-	-	-	-	C	Upstream variant			
15	54278947	Tnfrsf11b	rs33489239	A	G	-	-	-	-	G	Upstream variant			
15	54283277	Tnfrsf11b	rs262793811	G	A	-	-	-	-	A	Upstream variant			
15	54283285	Tnfrsf11b	rs230687316	A	G	-	-	-	-	G	Upstream variant			
15	54283374	Tnfrsf11b	rs244592538	T	G	-	-	-	-	G	Upstream variant			
15	54406313	Colec10	rs32088480	T	A	-	A	-	-	A	Upstream variant			
15	54406837	Colec10	rs32500070	T	C	-	C	-	-	C	upstream_variant			
15	54407117	Colec10	rs31622803	G	C	-	C	-	-	C	Upstream variant			
15	54409729	Colec10	rs33480153	C	A	-	A	-	-	A	Upstream variant			
15	54409899	Colec10	rs33479083	C	A	-	A	-	-	A	Upstream variant			
15	54410005	Colec10	rs32301243	C	T	-	T	-	-	T	Upstream variant			
15	54567323	Mal2	rs51841138	C	A	-	A	-	-	A	Upstream variant			
15	54568155	Mal2	rs32142140	G	A	-	A	-	-	A	Upstream variant			
15	54845847	Enpp2	rs6411953	T	C	-	C	-	-	C	Missense variant	N/D	743	1
15	54921351	Enpp2	rs31919117	C	G	-	G	-	-	G	Upstream variant			
15	54925070	Enpp2	rs244071159	T	C	-	C	-	-	C	Upstream variant			
15	55220217	**Deptor**	rs32271813	G	A	-	A	-	-	A	Missense variant	E/D	15	0.12
15	63825102	**Gsdmc2**	rs252605414	G	-	-	-	C	-	C	Missense variant	L/V	407	0.01
15	63848572	Gsdmc2	rs231708781	G	-	-	-	T *	-	T *	Upstream variant			
15	63849657	Gsdmc2	rs51282393	C	-	-	-	T *	-	T *	Upstream variant			
15	63873431	Gsdmc3	rs587096509	A	-	-	-	G	-	G	Upstream variant			
15	63905408	Gsdmc4	rs387408365	G	-	-	-	C	-	C	Upstream variant			
15	63913637	Gsdmc4	rs32238759	G	-	-	-	C	-	C	Upstream variant			
15	63915285	Gsdmc4	rs583638710	C	-	-	-	T *	-	T *	Upstream variant			

Chr: chromosome; Position: in bp; dbSNP: Single nucleotide polymorphism database; Ref: Reference or C57BL/6J SNP; Csq: SNP consequences. AA: Amino acid; AA coord: Amino acid coordinate. SIFT, Sorting Intolerant from Tolerant (intolerant SNP is highlighted in bold). “-” same as reference SNP. Not all upstream variants were shown due to space limitation. * Multiple consequences.

## Data Availability

All data reported in this article are included in Supplemental Materials and also through this link: https://figshare.com/s/7c96b96f6bb4a8dacd7f (accessed on 14 February 2022).

## Supplementary Materials

The following supporting information can be downloaded at: https://www.mdpi.com/article/10.3390/genes13030510/s1: Original data for R/qtl and Map Manager QTX analysis; Suppl Table on Positional candidate genes for metabolic trait QTL on chromosome 15 identified by haplotype analysis.
